# Molecular Detection and Genotypic Characterization of *Toxoplasma gondii* in Paraffin-Embedded Fetoplacental Tissues of Women with Recurrent Spontaneous Abortion 

**DOI:** 10.22074/ijfs.2016.4569

**Published:** 2016-11-01

**Authors:** Amir Abdoli, Abdolhossein Dalimi, Haleh Soltanghoraee, Fatemeh Ghaffarifar

**Affiliations:** 1 Department of Parasitology, Faculty of Medical Sciences, Tarbiat Modares University, Tehran, Iran; 2 Reproductive Biotechnology Research Center, Avicenna Research Institute, ACECR, Tehran, Iran

**Keywords:** *Toxoplasma gondii*, Abortion, Molecular Detection, Genotype, Iran

## Abstract

**Background:**

Congenital toxoplasmosis is an important cause of spontaneous abortion worldwide. However, there is limited information on detection and genotypic characterization of *Toxoplasma gondii (T. gondii)* in women with recurrent spontaneous abortion (RSA). The aim of this study is the molecular detection and genotypic characterization of *T. gondii* in formalin-fixed, paraffin-embedded fetoplacental tissues (FFPTs) of women with RSA that have referred to the Avicenna Research Institute in Tehran, Iran.

**Materials and Methods:**

This experimental research was undertaken on 210 FFPTs of women with RSA. The information of the patients was collected from the archives of Avicenna Research Institute in Tehran, Iran. After DNA extraction, the presence of T. gondii was examined by nested polymerase chain reaction targeting the GRA6 gene. Genotyping was performed on positive samples using polymerase chain reaction-restriction fragment length polymorphism (PCR-RFLP) that targeted the *GRA6* and *SAG3* genes. Sequencing was conducted on two GRA6 positive samples.

**Results:**

*T. gondii* DNA was detected in 3.8% (8/210) of the samples. Genotyping showed that all positive samples belonged to type III of the *T. gondii* genotype. Sequencing two genomic DNAs of the GRA6 gene revealed 99% similarity with each other and 99-100% similarity with *T. gondii* sequences deposited in GenBank. There were six patients with histories of more than three abortions; one patient had a healthy girl and another patient had two previous abortions. Abortions occurred in the first trimester of pregnancy in seven patients and in the second trimester of pregnancy in one patient.

**Conclusion:**

The results of this study have indicated that genotype III is the predominant type of *T. gondii* in women with RSA in Tehran, Iran. Also, our findings suggest that toxoplasmosis may play a role in the pathogenesis of RSA. However, further studies are needed to elucidate a clear relationship between *T. gondii* infection and RSA.

## Introduction

Toxoplasmosis is one of the most common parasitic diseases where approximately one-third of the world’s population is affected (1, 2). Approximately 25 to 30% of the world’s population is infected by *Toxoplasma gondii* (*T. gondii*). Nevertheless, the most common form of infection is asymptomatic (2-4). Human infections generally occur by the consumption of undercooked meatthat contains tissue cysts or by water and food contaminated with oocysts present in cat feces. Congenital infection is one of the most important sequels of toxoplasmosis in pregnant women (1). Congenital transmission of *T. gondii* predominantly occurs at the first time during pregnancy (3, 5). The approximate incidence rate of congenital toxoplasmosis is 1.5 cases per 1000 live births with a global incidence rate of 190,100 cases annually (6). Frequency of transplacental transmission and severity of congenital toxoplasmosis correlates with the gestational age of infected mothers. The highest rates of transplacental transmission occur in the third trimester of pregnancy; which usually results in asymptomatic infections at birth. However, they may develop clinical signs (such as chorioretinitis, slower mental and neurological development) at a later age (1). On the other hand, the severity of congenital toxoplasmosis is highest in the first and second trimesters of pregnancy which usually results in abortion or stillbirth (1, 5, 7, 8).

Recurrent spontaneous abortion (RSA) is the loss of three or more consecutive pregnancies before 20 weeks of pregnancy (9) and affects approximately 1 to 2% of couples trying to conceive (10). Several factors-genetic background, anatomical abnormalities, endocrine disruption, autoimmune disorder, and infectious diseases have been attributed to play roles in the etiology of RSA (9-11). Infectious agents account for 0.5 to 5% of RSA (10). The most common infectious causes of RSA are *Chlamydia trachomatis, Ureaplasmaurealyticum, Mycoplasma hominis,* cytomegalovirus (CMV), and human papillomaviruses (HPV) (9, 11). Several studies have reported significantly higher seroprevalence of ToRCH infections in women with spontaneous abortion or negative obstetric history, including preterm deliveries, intrauterine deaths or growth retardation (12-16).

Although several studies have reported an association between *T. gondii* infection and spontaneous abortion (6), the role of toxoplasmosis in the etiology of RSA is less clear. Hence, we have investigated the rate of *T. gondii* infection in formalin-fixed, paraffin-embedded fetoplacental tissues (FFPTs) of women with RSA that referred to the Avicenna Research Institute in Tehran, Iran.

## Materials and Methods

This experimental study was performed on archived FFPTs of women with RSA that referred to the Avicenna Research Institute in Tehran, Iran during 2013-2015. This study was approved by the Ethical Committees of Tarbiat Modares University and Avicenna Research Institute. Avicenna Research Institute was consent about the research on the archived FFPTs of women.

### Patients and samples

We collected 210 FFPTs of aborted fetuses or placentas of women with recurrent abortion from the archives of the Avicenna Research Institute in Tehran, a referral center for infertile couples in Iran. Information of clinical symptoms, pathological findings, and genetic background were obtained from patients’ medical records.

### DNA extraction

For each FFPT, five 10 μm thick sections were cut and transferred to 1.5 ml microtubes. In order to avoid cross-contamination, we used a new, sterile disposable microtome blade for each block. Sections were deparaffinized by the addition of 1 ml xylene (Merck, Germany) for 15 minutes at 50°C. Subsequently, the tubes were centrifuged at 13000 g for 5 minutes and the supernatant was discarded. This step was repeated twice. The samples were rehydrated in a descending ethanol series (100, 90, 80, 70%) and subsequently washed with distilled water. For DNA extraction, 800 μL of lysis buffer (50 mM tris-HCl, pH=8.0, 25 mM EDTA, and 400 mM NaCl), 100 μL 10% sodium dodecyl sulfate (SDS, Merck, Germany), and 10 μL proteinase K (20 μg/μL, Thermo Fisher Scientific, Wilmington, DE, USA) were added to each tube (17). The suspension was incubated at 55ºC for 72 hours. After overnight, an additional of 10 μL proteinase K (20 μg/μL) was added to each tube (18). In order to precipitate undissolved proteins and debris, we added 300 μL of 6 M NaCl to each tube for 15 minutes at 4°C. After centrifugation (13000 g for 15 minutes), the supernatant was transferred to 1.5 ml microtubes (17). Then, 800 μl of phenol-chloroform-isoamyl alcohol (25:24:1) was added to each microtube. The microtubes were centrifuged (13000 g for 15 minutes) and we transferred the supernatants to new microtubes. Subsequently, 1 ml of chloroform was added to each microtube. The microtubes were centrifuged (13000 g for 15 minutes) and the supernatant was transferred to sterile microtubes. DNA was precipitated by the addition of one-tenth the volume of a sodium acetate solution (3 M, pH=5.2) and twice the volume of cold 100% ethanol, kept at -20°C overnight, and subsequently centrifuged at 13000 g for 20 minutes. Finally, the pellet was washed with 70% ethanol, centrifuged (13000 g for 15 minutes), resuspended in 50 μL of distilled water, and stored at -20°C until use.In order to ensure that the DNA was extracted, we used two *T. gondii* positive tissue samples (GenBank accession numbers. KR809554 and KR809555) which had been detected in our previous study (19). These positive samples were fixed in formalin and embedded in paraffin after which the following procedure for DNA extraction was performed. We also used the Rh strain of *T. gondii* as a positive control.

### Detection of *T. gondii* infection by nested polymerase chain reaction

PCR was conducted using a pair of *T. gondii*-specific primers:

*GAR6*-F1: 5'-ATTTGTGTTTCCGAGCAGGT-3' and R1: 5'-GCACCTTCGCTTGTGGTT-3'.

Nested-PCR was performed with primers:

*GAR6*-F2: 5'-TTTCCGAGCAGGTGACCT-3' and R2: 5'-TCGCCGAAGAGTTGACATAG-3' (20).

Amplifications were conducted ina final volume of a 20 μL reaction mixturethat contained 10 μL of 2x Taq DNA polymerase Master Mix with 2 mM MgCl_2_ (Cat. no. A170301, Ampliqon, Denmark), 10 pmol of each primer, 5 μL of distilled water, and 3 μL of template DNA. For nested-PCR, one μL of the first round PCR product was used as the template. For each reaction, two positive controls (DNA extracted from *T. gondii* paraffin-embedded tissuesand the RH strain of *T. gondii*) and a negative control (double distilled water) were included. Amplification was performed with initial denaturation for 5 minutes at 95°C, followed by 35 cycles at 95°C for 30 seconds (denaturation), annealing at 59°C in the first round, and 57°C in nested PCR for 30 seconds, extension at 72°C for 30 seconds, and final extension at 72°C for 10 minutes. A total of 5 μl of nested-PCR products along with a 100-bp DNA ladder were electrophoresed in 1.5% safe stain (Sinaclon, Iran) agarose gels and visualized under ultra-violet trans-illumination.

### Genotyping of positive samples by restriction fragment length polymorphism

Positive samples were genotyped using *GRA6* and *SAG3* markers (21). First, we digested the nested-PCR products of GRA6 positive samples using Tru1I (MseI) restriction enzyme (Cat. No. ER0982, Thermo Fisher Scientific, USA) as previously described (20). Digestion was conducted in a final volume of 16 μL reaction mixtures that contained 5 μL of the nested-PCR products, 1μL of Tru1I enzyme, 1 μL of 10X Buffer R, and 9 μL of nuclease-free water. Then, the reaction mixtures were incubated at 65°C for 1 hour according to the manufacturer’s instructions. A total of 10 μl of restriction fragments were electrophoresed by Tris-acetate-EDTA (TAE) buffer through 3% (w/v) agarose gel stained with safe stain and visualized under UV transillumination. We conducted genotyping of the positive samples by the *SAG3* marker (21, 22). Nested-PCR was carried out for positive samples using the *SAG3* marker as previously described (22). Next, the products were digested using BcnI (NciI) restriction enzyme (Cat. No. ER0061, Thermo Fisher Scientific, USA) at 37°C for 6 hours according to the manufacturer’s protocols. The restriction fragments were electrophoresed and visualized under UV transillumination. The type of *T. gondii* was determined according to the restriction patterns after digestion with restriction enzymes (21). In order to determine better illustrationpatterns of the genotypes, the *GRA6* and *SAG3* sequences of three types of *T. gondii* (RH type I, ME49 type II, and NED type III) were obtained fromGenBank and digested by their restriction enzymes using NEBcutter V2.0 (http://nc2.neb.com/NEBcutter2/).

### Nucleotide sequence analysis of the GRA6 gene

We extracted two positive nested-PCR products of the GRA6 gene from the gel (Vivantis Gel Purification kit, Selangor DarulEhsan, Malaysia) according to the manufacturer’s instructions. The products were sequenced in the forward and reverse directions by the Sequetech Corporation (Mountain View, CA, USA), edited with BioEdit software, (23) and compared with GRA6 partial sequences of *T. gondii* available in GenBank.

## Results

### Detection of *T. gondii* in women with recurrent spontaneous abortion

**Fig.1:**
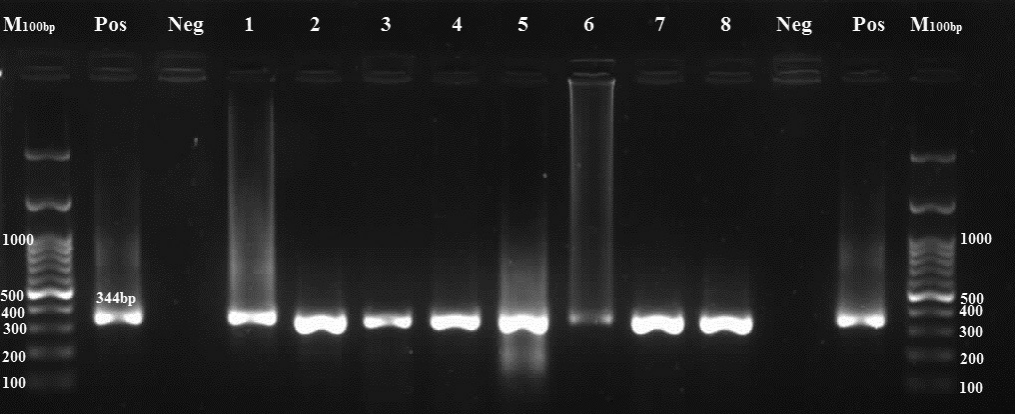
PCR products of the GRA6 positive samples. *Toxoplasma gondii (T. gondii)* positive samples give a 344-bp band. M; 100 bp DNA marker, Pos; Positive control, Neg; Negative control, and Lanes 1-8; Positive samples.

*T. gondii* DNA was detected in 8 out of 210 samples (3.8%) by the GRA6 marker (Fig .1).

As shown in Table 1, patients had a mean age of 33.5 years (range: 28-39 years). There were seven patients with a history of previous abortion (patients 1-3, 5-8). From these, six occurred in the first trimester and one occurred in the second trimester (patient 1). One patient had a healthy girl (patient 4) with no history of previous abortion. The abortion of this patient (patient 4) was occured in the first trimester of pregnancy. Patient 1had clinical symptoms of fever and severe necrotizing chorioamnionitis before the abortion. Patient 3 reported clinical symptoms such as rapid heart beat, maternal anemia, and edema of the legs and ankles before the abortion. The edema resolved after the abortion. Patient 2 had a history of hypothyroidism. Nonspecific symptoms were reported from other patients before the abortions (Table 1).

**Table 1:** Information of the *Toxoplasma gondii (T. gondii)* infected women with recurrent spontaneous abortion (RSA)


PatientNo.	Age (Y) CityProvince	Number of gestations (G),Number of abortions (AB)	Week of abortion	Chromosomal aneuploidies^§^	Pathological findings in fetoplacental tissues	Symptoms

1	36 Abhar Zanjan	G6, AB6All pregnancies aborted at second trimester	LMP^†^: 16w(Second trimester)	Not detected	Inflammatory cell infiltration with patchy amnionic necrotizing foci in the membrane	Fever, severe abdominal and back pain,premature rupture of membranes (PPROM)
2	31 Tuyserkan Hamedan	G4, AB4All pregnancies aborted at first trimester	LMP: 11w+2dUltrasound: 8w(First trimester)	Not detected	No remarkable pathological findings	Hypothyroidism
3	39 Eslamshahr Tehran	G8, AB8All pregnancies aborted at first trimester	LMP: 7w(First trimester)	Not detected	No remarkable pathological findings	No specific symptoms
4	38 Tehran Tehran	G2, AB1She has one healthy girl	LMP: 11w(First trimester)	Not detected	No remarkable pathological findings	Rapid heartbeat, anemia andedema of the legs and ankles
5	36 Karaj Alborz	G3, AB3All pregnancies aborted at first trimester.	Ultrasound: 6w+4d(First trimester)	MLPA findings compatible with an extra copy of chromosome 15 (trisomy 15)	The membrane showed calcification without inflammation	No specific symptoms
6	28 Kashan Isfahan	G5, AB5	LMP: 13wUltrasound: 11w(First trimester)	Not detected	No remarkable pathological findings	No specific symptoms
7	31 Tehran Tehran	G3, AB3All pregnancies aborted at first trimester	LMP: 11wUltrasound: 8w+3d(First trimester)	Not detected	No remarkable pathological findings	No specific symptoms
8	29 Tehran Tehran	G2, AB2All pregnancies aborted at first trimester	LMP: 11wUltrasound: 8w(First trimester)	Not detected	No remarkable pathological findings	No specific symptoms


^†^; LMP: Last menstrual period and ^§^; Chromosomal aneuploidies were detected using multiplex ligation-dependent probe amplification (MLPA).

### Genotyping of positive samples

**Fig.2:**
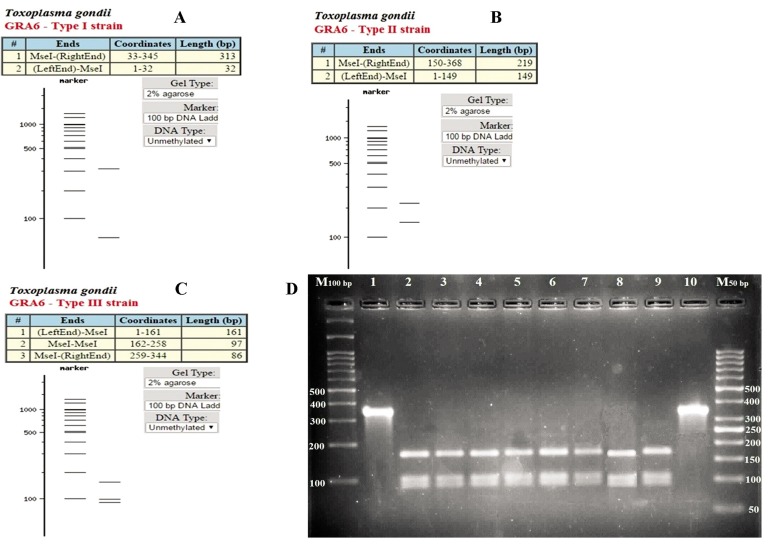
Genotyping of positive samples with the GRA6 marker. The products were digested with Tru1I enzyme. A, B, C. Patterns of three types of *Toxoplasma gondii (T. gondii)* genotype, and D. Agarose gel electrophoresis of PCR products digested with Tru1I enzyme. M; 100 and 50 bp DNA marker, Lanes 1 and 10; Undigested positive samples, and Lanes 2-9; *T. gondii* genotype type III.

**Fig.3:**
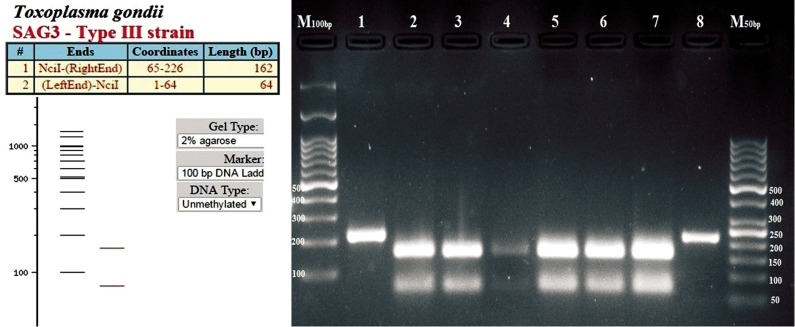
Genotyping of positive samples with the *SAG3* marker. The products were digested with BcnI enzyme. A. Patterns of *Toxoplasma gondii (T. gondii)* type III genotype and B. Agarose gel electrophoresis of PCR products digested with BcnI enzyme. M; 100 and 50 bp DNA marker, Lanes 1 and 8; Uundigested positive samples, and Lanes 2-7; *T. gondii* genotype type III.

GRA6 completely characterized eight samples as *T. gondii* genotype III (Fig .2). Genotyping of positive samples were conducted by using the *SAG3* marker. The results showed amplification of *SAG3* in six out of eight *GRA6* positive samples. Digestion of *SAG3* PCR products by BcnI enzyme determined that all six products belonged to *T. gondii* genotype III (Fig .3).

### Sequencing of the GRA6 gene

We submitted two GRA6 nucleotide sequences with a length of 344 bp to the GenBank database (KT735111, KT735112). The alignment of our sequences revealed the highest similarity (100%) with *T. gondii* isolated from cat (KP792620, KP792621), sheep (KT735113-19), rat (KP792610, KP792613), and bird (KR809554-8, KP792606-9, KP792600-2) hosts in Iran (Table 2).

**Table 2:** Multiple sequence alignment of the GRA6 gene of *Toxoplasma gondii (T. gondii)* from our samples and other hosts in Iran. Our samples are shown in red as accession numbers KT735111 and KT735112


KT809309	TTTCCGAGCAGGTGACCTGGGTCGCTTTTTTGAAACAGCAGGAAAACAGCTTCGTGGTGC
KP792604	TTTCCGAGCAGGTGACCTGGGTCGCTTTTTTGAAACAGCAGGAAAACAGCTT**T**GTGGTGC
KP792605	TTTCCGAGCAGGTGACCTGGGTCGCTTTTTTGAAACAGCAGGAAAACAGCTT**T**GTGGTGC
KR809555	TTTCCGAGCAGGTGACCTGGGTCGCTTTTTTGAAACAGCAGGAAAACAGCTTCGTGGT**T**C
**KT735112**	TTTCCGAGCAGGTGACCTGGGTCGCTTTTTTGAAACAGCAGGA**G**AACAGCTTCGTGGTGC
KP792614	TTTCCGAGCAGGTGACCTGGGTCGCTTTTTTGAAACAGCAGGAAAACAGCTTCGTGGTGC
KR809556	TTTCCGAGCAGGTGACCTGGGTCGC**C**TTTTTGAAACAGCAGGAAAACAGCTTCGTGGTGC
KR809558	TTTCCGAGCAGGTGACCTGGGTCGCTTTTTTG**T**AACAGCAGGAAAACAGCTTCGTGGTGC
**KT735111**	TTTCCGAGCAGGTGACCTGGGTCGCTTTTTTGAAACAGCAGGAAAACAGCTTCGTGGTGC
KT735113	TTTCCGAGCAGGTGACCTGGGTCGCTTTTTTGAAACAGCAGGAAAACAGCTTCGTGGTGC
KT735114	TTTCCGAGCAGGTGACCTGGGTCGCTTTTTTGAAACAGCAGGAAAACAGCTTCGTGGTGC
KT735115	TTTCCGAGCAGGTGACCTGGGTCGCTTTTTTGAAACAGCAGGAAAACAGCTTCGTGGTGC
KT735116	TTTCCGAGCAGGTGACCTGGGTCGCTTTTTTGAAACAGCAGGAAAACAGCTTCGTGGTGC
KT735117	TTTCCGAGCAGGTGACCTGGGTCGCTTTTTTGAAACAGCAGGAAAACAGCTTCGTGGTGC
KT735119	TTTCCGAGCAGGTGACCTGGGTCGCTTTTTTGAAACAGCAGGAAAACAGCTTCGTGGTGC
KR809557	TTTCCGAGCAGGTGACCTGGGTCGCTTTTTTGAAACAGCAGGAAAACAGCTTCGTGGTGC
KR809554	TTTCCGAGCAGGTGACCTGGGTCGCTTTTTTGAAACAGCAGGAAAACAGCTTCGTGGTGC
KP792621	TTTCCGAGCAGGTGACCTGGGTCGCTTTTTTGAAACAGCAGGAAAACAGCTTCGTGGTGC
KP792620	TTTCCGAGCAGGTGACCTGGGTCGCTTTTTTGAAACAGCAGGAAAACAGCTTCGTGGTGC
KP792609	TTTCCGAGCAGGTGACCTGGGTCGCTTTTTTGAAACAGCAGGAAAACAGCTTCGTGGTGC
KP792610	TTTCCGAGCAGGTGACCTGGGTCGCTTTTTTGAAACAGCAGGAAAACAGCTTCGTGGTGC
KT735118	T͙T͙T͙C͙C͙G͙A͙G͙C͙A͙G͙G͙T͙G͙A͙C͙C͙T͙G͙G͙G͙T͙C͙G͙C͙ṬT͙T͙T͙T͙T͙G͙A͙A͙A͙C͙A͙G͙C͙A͙G͙G͙A͙ẠA͙A͙C͙A͙G͙C͙T͙T͙C̣G͙T͙G͙G͙T͙G̣C͙

KT809309	CACGTAGCGTGCTTGTTGGCGACTACCTTTTTTTCTTGGGAGTGTCGGCGAAATGGCACA
KP792604	CACGTAGCGTGCTTGTTGGCGACTACCTTTTTTTCTTGGGAGTGTCGGCGAAATGGCACA
KP792605	CACGTAGCGTGCTTGTTGGCGACTACCTTTTTTTCTTGGGAGTGTCGGCGAAATGGCACA
KR809555	CACGTAGCGTGCTTGTTGGCGACTACCTTTTTTTCTTGGGAGTGTCGGCGAAATGGCACA
**KT735112**	CACGTAGCGTGCTTGTTGGCGACTACCTTTTTTTCTTGGGAGTGTCGGCGAAATGGCACA
KP792614	CACGTAGCGTGCTTGTTGGCGACTACCTTTTTTTCTTGGGAGTGTCGGCGAAATGGCACA
KR809556	CACGTAGCGTGCTTGTTGGCGACTACCTTTTTTTCTTGGGAGTGTCGGCGAAATGGCACA
KR809558	CACGTAGCGTGCTTGTTGGCGACTACCTTTTTTTCTTGGGAGTGTCGGCGAAATGGCACA
**KT735111**	CACGTAGCGTGCTTGTTGGCGACTACCTTTTTTTCTTGGGAGTGTCGGCGAAATGGCACA
KT735113	CACGTAGCGTGCTTGTTGGCGACTACCTTTTTTTCTTGGGAGTGTCGGCGAAATGGCACA
KT735114	CACGTAGCGTGCTTGTTGGCGACTACCTTTTTTTCTTGGGAGTGTCGGCGAAATGGCACA
KT735115	CACGTAGCGTGCTTGTTGGCGACTACCTTTTTTTCTTGGGAGTGTCGGCGAAATGGCACA
KT735116	CACGTAGCGTGCTTGTTGGCGACTACCTTTTTTTCTTGGGAGTGTCGGCGAAATGGCACA
KT735117	CACGTAGCGTGCTTGTTGGCGACTACCTTTTTTTCTTGGGAGTGTCGGCGAAATGGCACA
KT735119	CACGTAGCGTGCTTGTTGGCGACTACCTTTTTTTCTTGGGAGTGTCGGCGAAATGGCACA
KR809557	CACGTAGCGTGCTTGTTGGCGACTACCTTTTTTTCTTGGGAGTGTCGGCGAAATGGCACA
KR809554	CACGTAGCGTGCTTGTTGGCGACTACCTTTTTTTCTTGGGAGTGTCGGCGAAATGGCACA
KP792621	CACGTAGCGTGCTTGTTGGCGACTACCTTTTTTTCTTGGGAGTGTCGGCGAAATGGCACA
KP792620	CACGTAGCGTGCTTGTTGGCGACTACCTTTTTTTCTTGGGAGTGTCGGCGAAATGGCACA
KP792609	CACGTAGCGTGCTTGTTGGCGACTACCTTTTTTTCTTGGGAGTGTCGGCGAAATGGCACA
KP792610	CACGTAGCGTGCTTGTTGGCGACTACCTTTTTTTCTTGGGAGTGTCGGCGAAATGGCACA
KT735118	C͙A͙C͙G͙T͙A͙G͙C͙G͙T͙G͙C͙T͙T͙G͙T͙T͙G͙G͙C͙G͙A͙C͙T͙A͙C͙C͙T͙T͙T͙T͙T͙T͙T͙C͙T͙T͙G͙G͙G͙A͙G͙T͙G͙T͙C͙G͙G͙C͙G͙A͙A͙A͙T͙G͙G͙C͙A͙C͙A͙

KT809309	CGGTGGCATCCATCTGAGGCAGAAGCGTAACT**C**CTGTCCTTTAACTGTCTCCACAGTTGC
KP792604	CGGTGGCATCCATCTGAGGCAGAAGCGTAACTTCTGTCCTTTAACTGTCTCCACAGTTGC
KP792605	CGGTGGCATCCATCTGAGGCAGAAGCGTAACTTCTGTCCTTTAACTGTCTCCACAGTTGC
KR809555	CGGTGGCATCCATCTGAGGCAGAAGCGTAACTTCTGTCCTTTAACTGTCTCCACAGTTGC
**KT735112**	CGGTGGCA**C**CCATCTGAGGCAGAAGCGTAACTTCTGTCCTTTAACTGTCTCCACAGTTGC
KP792614	CGGTGGCATCCATCTGAGGCAGAAGCGTAACTTCTGTCCTTTAACTGTCTCCACAGTTGC
KR809556	CGGTGGCATCCATCTGAGGCAGAAGCGTAACTTCTGTCCTTTAACTGTCTCCACAGTTGC
KT735112	TGTGGTCTTTGTAGTTTTCATGGGTGTACTCGTCAATTCGTTGGGTGGAGTCGCTGTCGC
KP792614	CGGTGGCATCCATCTGAGGCAGAAGCGTAACTTCTGTCCTTTAACTGTCTCCACAGTTGC
KR809556	CGGTGGCATCCATCTGAGGCAGAAGCGTAACTTCTGTCCTTTAACTGTCTCCACAGTTGC
KR809558	CGGTGGCATCCATCTGAGGCAGAAGCGTAACTTCTGTCCTTTAACTGTCTCCACAGTTGC
**KT735111**	CGGTGGCATCCATCTGAGGCAGAAGCGTAACTTCTGTCCTTTAACTGTCTCCACAGTTGC
KT735113	CGGTGGCATCCATCTGAGGCAGAAGCGTAACTTCTGTCCTTTAACTGTCTCCACAGTTGC
KT735114	CGGTGGCATCCATCTGAGGCAGAAGCGTAACTTCTGTCCTTTAACTGTCTCCACAGTTGC
KT735115	CGGTGGCATCCATCTGAGGCAGAAGCGTAACTTCTGTCCTTTAACTGTCTCCACAGTTGC
KT735116	CGGTGGCATCCATCTGAGGCAGAAGCGTAACTTCTGTCCTTTAACTGTCTCCACAGTTGC
KT735117	CGGTGGCATCCATCTGAGGCAGAAGCGTAACTTCTGTCCTTTAACTGTCTCCACAGTTGC
KT735119	CGGTGGCATCCATCTGAGGCAGAAGCGTAACTTCTGTCCTTTAACTGTCTCCACAGTTGC
KR809557	CGGTGGCATCCATCTGAGGCAGAAGCGTAACTTCTGTCCTTTAACTGTCTCCACAGTTGC
KR809554	CGGTGGCATCCATCTGAGGCAGAAGCGTAACTTCTGTCCTTTAACTGTCTCCACAGTTGC
KP792621	CGGTGGCATCCATCTGAGGCAGAAGCGTAACTTCTGTCCTTTAACTGTCTCCACAGTTGC
KP792620	CGGTGGCATCCATCTGAGGCAGAAGCGTAACTTCTGTCCTTTAACTGTCTCCACAGTTGC
KP792609	CGGTGGCATCCATCTGAGGCAGAAGCGTAACTTCTGTCCTTTAACTGTCTCCACAGTTGC
KP792610	CGGTGGCATCCATCTGAGGCAGAAGCGTAACTTCTGTCCTTTAACTGTCTCCACAGTTGC
KT735118	C͙G͙G͙T͙G͙G͙C͙A͙ṬC͙C͙A͙T͙C͙T͙G͙A͙G͙G͙C͙A͙G͙A͙A͙G͙C͙G͙T͙A͙A͙C͙T͙ṬC͙T͙G͙T͙C͙C͙T͙T͙T͙A͙A͙C͙T͙G͙T͙C͙T͙C͙C͙A͙C͙A͙G͙T͙**C̣**G͙C͙

KT809309	TGTGGTCTTTGTAGTTTTCATGGGTGTACTCGTCAATTCGTTGGGTGGAGTCGC**C**GTCGC
KP792604	TGTGGTCTTTGTAGTTTTCATGGGTGTACTCGTCAATTCGTTGGGTGGAGTCGCTGTCGC
KP792605	TGTGGTCTTTGTAGTTTTCATGGGTGTACTCGTCAATTCGTTGGGTGGAGTCGCTGTCGC
KR809555	TGTGGTCTTTGTAGTTTTCATGGGTGTACTCGTCAATTCGTTGGGTGGAGTCGCTGTCGC
**KT735112**	TGTGGTCTTTGTAGTTTTCATGGGTGTACTCGTCAATTCGTTGGGTGGAGTCGCTGTCGC
KP792614	TGTGGTCTTTGTAGTTTTCATGGGTGTACTCGTCAATTCGTTGGGTGGAGTCGCTGTCGC
KR809556	TGTGGTCTTTGTAGTTTTCATGGGTGTACTCGTCAATTCGTTGGGTGGAGTCGCTGTCGC
KR809558	TGTGGTCTTTGTAGTTTTCATGGGTGTACTCGTCAATTCGTTGGGTGGAGTCGCTGTCGC
**KT735111**	TGTGGTCTTTGTAGTTTTCATGGGTGTACTCGTCAATTCGTTGGGTGGAGTCGCTGTCGC
KT735113	TGTGGTCTTTGTAGTTTTCATGGGTGTACTCGTCAATTCGTTGGGTGGAGTCGCTGTCGC
KT735114	TGTGGTCTTTGTAGTTTTCATGGGTGTACTCGTCAATTCGTTGGGTGGAGTCGCTGTCGC
KT735115	TGTGGTCTTTGTAGTTTTCATGGGTGTACTCGTCAATTCGTTGGGTGGAGTCGCTGTCGC
KT735116	TGTGGTCTTTGTAGTTTTCATGGGTGTACTCGTCAATTCGTTGGGTGGAGTCGCTGTCGC
KT735117	TGTGGTCTTTGTAGTTTTCATGGGTGTACTCGTCAATTCGTTGGGTGGAGTCGCTGTCGC
KT735119	TGTGGTCTTTGTAGTTTTCATGGGTGTACTCGTCAATTCGTTGGGTGGAGTCGCTGTCGC
KR809557	TGTGGTCTTTGTAGTTTTCATGGGTGTACTCGTCAATTCGTTGGGTGGAGTCGCTGTCGC
KR809554	TGTGGTCTTTGTAGTTTTCATGGGTGTACTCGTCAATTCGTTGGGTGGAGTCGCTGTCGC
KP792621	TGTGGTCTTTGTAGTTTTCATGGGTGTACTCGTCAATTCGTTGGGTGGAGTCGCTGTCGC
KP792620	TGTGGTCTTTGTAGTTTTCATGGGTGTACTCGTCAATTCGTTGGGTGGAGTCGCTGTCGC
KP792609	TGTGGTCTTTGTAGTTTTCATGGGTGTACTCGTCAATTCGTTGGGTGGAGTCGCTGTCGC
KP792610	TGTGGTCTTTGTAGTTTTCATGGGTGTACTCGTCAATTCGTTGGGTGGAGTCGCTGTCGC
KT735118	T͙G͙T͙G͙G͙T͙C͙T͙T͙T͙G͙T͙A͙G͙T͙T͙T͙T͙C͙A͙T͙G͙G͙G͙T͙G͙T͙A͙C͙T͙C͙G͙T͙C͙A͙A͙T͙T͙C͙G͙T͙T͙G͙G͙G͙T͙G͙G͙A͙G͙T͙C͙G͙C͙ṬG͙T͙C͙G͙C͙

KT809309	AGCAGACAGCGATGGTGTTAAGCAGACCCCTTCGGAAACCGGTTCGAGCGG**G**GGACAGCA
KP792604	AGCAGACAGCGATGGTGTTAAGCAGACCCCTTCGGAAACCGGTTCGAGCGGTGGACAGCA
KP792605	AGCAGACAGCGATGGTGTTAAGCAGACCCCTTCGGAAACCGGTTCGAGCGGTGGACAGCA
KR809555	AGCAGACAGCG**G**TGGTGTTAAGCAGACCCCTTCGGAAACCGGTTCGAGCGGTGGACAGCA
**KT735112**	AGCAGACAGCGATGGTGTTAAGCAGACCCCTTCGGAAACCGGTTCGAGCGGTGGACAGCA
KP792614	AGCAGACAGCGATGGTGTTAAGCAG**G**CCCCTTCGGAAACCGGTTCGAGCGGTGGACAGCA
KR809556	AGCAGACAGCGATGGTGTTAAGCAGACCCCTTCGGAAACCGGTTCGAGCGGTGGACAGCA
KR809558	AGCAGACAGCGATGGTGTTAAGCAGACCCCTTCGGAAACCGGTTCGAGCGGTGGACAGCA
**KT735111**	AGCAGACAGCGATGGTGTTAAGCAGACCCCTTCGGAAACCGGTTCGAGCGGTGGACAGCA
KT735113	AGCAGACAGCGATGGTGTTAAGCAGACCCCTTCGGAAACCGGTTCGAGCGGTGGACAGCA
KT735114	AGCAGACAGCGATGGTGTTAAGCAGACCCCTTCGGAAACCGGTTCGAGCGGTGGACAGCA
KT735115	AGCAGACAGCGATGGTGTTAAGCAGACCCCTTCGGAAACCGGTTCGAGCGGTGGACAGCA
KT735116	AGCAGACAGCGATGGTGTTAAGCAGACCCCTTCGGAAACCGGTTCGAGCGGTGGACAGCA
KT735117	AGCAGACAGCGATGGTGTTAAGCAGACCCCTTCGGAAACCGGTTCGAGCGGTGGACAGCA
KT735119	AGCAGACAGCGATGGTGTTAAGCAGACCCCTTCGGAAACCGGTTCGAGCGGTGGACAGCA
KR809557	AGCAGACAGCGATGGTGTTAAGCAGACCCCTTCGGAAACCGGTTCGAGCGGTGGACAGCA
KR809554	AGCAGACAGCGATGGTGTTAAGCAGACCCCTTCGGAAACCGGTTCGAGCGGTGGACAGCA
KP792621	AGCAGACAGCGATGGTGTTAAGCAGACCCCTTCGGAAACCGGTTCGAGCGGTGGACAGCA
KP792620	AGCAGACAGCGATGGTGTTAAGCAGACCCCTTCGGAAACCGGTTCGAGCGGTGGACAGCA
KP792609	AGCAGACAGCGATGGTGTTAAGCAGACCCCTTCGGAAACCGGTTCGAGCGGTGGACAGCA
KP792610	AGCAGACAGCGATGGTGTTAAGCAGACCCCTTCGGAAACCGGTTCGAGCGGTGGACAGCA
KT735118	A͙G͙C͙A͙G͙A͙C͙A͙G͙C͙G͙ẠT͙G͙G͙T͙G͙T͙T͙A͙A͙G͙C͙A͙G͙ẠC͙C͙C͙C͙T͙T͙C͙G͙G͙A͙A͙A͙C͙C͙G͙G͙T͙T͙C͙G͙A͙G͙C͙G͙G͙ṬG͙G͙A͙C͙A͙G͙C͙A͙

KT809309	AGAAGCAGTGGGGACCACTGAAGACTATGTCAACTCTTCGGCGA
KP792604	AGAAGCAGTGGGGACC**C**CTGAAGACTATGTCAACTCTTCGGCGA
KP792605	AGAAGCAGTGGGGACCACTGAAGACTATGTCAACTCTTCGGCGA
KR809555	AGAAGCAGTGGGGACCACTGAAGACTATGTCAACTCTTCGGCGA
**KT735112**	AGAAGCAGTGGGGACCACTGAAGACTATGTCAACTCTTCGGCGA
KP792614	AGAAGCAGTGGGGACCACTGAAGACTATGTCAACTCTTCGGCGA
KR809556	AGAAGCAGTGGGGACCACTGAAGACTATGTCAACTCTTCGGCGA
KR809558	AGAAGCAGTGGGGACCACTGAAGACTATGTCAACTCTTCGGCGA
**KT735111**	AGAAGCAGTGGGGACCACTGAAGACTATGTCAACTCTTCGGCGA
KT735113	AGAAGCAGTGGGGACCACTGAAGACTATGTCAACTCTTCGGCGA
KT735114	AGAAGCAGTGGGGACCACTGAAGACTATGTCAACTCTTCGGCGA
KT735115	AGAAGCAGTGGGGACCACTGAAGACTATGTCAACTCTTCGGCGA
KT735116	AGAAGCAGTGGGGACCACTGAAGACTATGTCAACTCTTCGGCGA
KT735117	AGAAGCAGTGGGGACCACTGAAGACTATGTCAACTCTTCGGCGA
KT735119	AGAAGCAGTGGGGACCACTGAAGACTATGTCAACTCTTCGGCGA
KR809557	AGAAGCAGTGGGGACCACTGAAGACTATGTCAACTCTTCGGCGA
KR809554	AGAAGCAGTGGGGACCACTGAAGACTATGTCAACTCTTCGGCGA
KP792621	AGAAGCAGTGGGGACCACTGAAGACTATGTCAACTCTTCGGCGA
KP792620	AGAAGCAGTGGGGACCACTGAAGACTATGTCAACTCTTCGGCGA
KP792609	AGAAGCAGTGGGGACCACTGAAGACTATGTCAACTCTTCGGCGA
KP792610	AGAAGCAGTGGGGACCACTGAAGACTATGTCAACTCTTCGGCGA
KT735118	A͙G͙A͙A͙G͙C͙A͙G͙T͙G͙G͙G͙G͙A͙C͙C͙ẠC͙T͙G͙A͙A͙G͙A͙C͙T͙A͙T͙G͙T͙C͙A͙A͙C͙T͙C͙T͙T͙C͙G͙G͙C͙G͙A͙


*; Exact match between all sequences and .; Mismatch with at least one sequence.

## Discussion

In the current study, we detected *T. gondii* DNA in 3.8% of women with RSA. To our knowledge, this study was the first report of molecular diagnosis of *T. gondii* in women with RSA. In previous studies in Iran, Ghasemi et al. (8, 24) detected *T. gondii* DNA in 7.3% (6/82) of women with spontaneous abortion and in 3.6% (1/28) of women with stillbirth in Tehran. Asgari et al. (25) detected *T. gondii* DNA in 14.4% (78/542) of paraffin-embedded tissue samples from women with spontaneous abortion in Shiraz, Southern Iran. Hoveyda et al. (26) detected *T. gondii* DNA in 15.48% (10/65) of paraffin-embedded tissue samples from Iranian women with abortions by PCR. Genotyping of positive samples by PCR-restriction fragment length polymorphism (RFLP) has indicated that all eight positive samples belonged to genotype III of *T. gondii*. is classified into three main genotypes (type I, II, and III) with some differences in virulence and epidemiological patterns (27, 28). Genotype III is the most prevalenttype of *T. gondii* worldwide (27, 29). However, type I has the highest virulence of among *T. gondii* genotypes (28). In Iran, genotype III is the most prevalent type of *T. gondii* (30), however genotype II (30, 31) and in some studies genotype I has been reported in different hosts (32, 33).

Association of *T. gondii* seropositivity with infertility or bad obstetric outcomes has been reported in different studies. In this regard, El-Tantawy et al. (34) observed significantly higher seroprevalcenc of *T. gondii* in infertile women. In that study, 61.85% (193/312) of infertile and 44% (44/100) of fertile women had *T. gondii* IgG seropositivity in Egypt. Malik et al. (35) demonstrated a significantly higher seroprevalence of *T. gondii* in 417 women with unfavorable obstetric history such as intrauterine deaths, intrauterine growth retardation, and preterm deliveries in India. According to the results, *T. gondii* IgM antibody was detected in 28% (120/417) of women with negative obstetric history, which 57% (68/120) had a history of previous abortion. Interestingly, *T. gondii* IgM antibody was found in 76.5% of women with two or more abortions and 23.5% of women with a single abortion. Toxoplasmosis was diagnosed in 6 out of 9 (66.7%) patients with secondary infertility and 3 (33.3%) with primary infertility (35). Aral et al. (36) did not find a significant association between *T. gondii* seropositivity and infertility in women in Turkey.

In recent years, several studies were conducted about the influences of latent (asymptomatic) toxoplasmosis on mothers and their offspring (3, 4, 37). In this regard, Kaňková and Flegr (38) reported that pregnant women with latent toxoplasmosis (IgG seropositive women) had developmentally younger fetuses (based on ultrasound scan) comparedto *T. gondii* negative women at week 16 of pregnancy. Kaňková et al. (39) also demonstrated that infants of mothers with latent toxoplasmosis had significantly slower postnatal motor development than mothers without latent toxoplasmosis during the first year of life. Another study by the same group revealed that *T. gondii*-infected pregnant women had used significantly more assisted reproductive technology to conceive compared to *T. gondii*-negative women. *T. gondii*-infected women had a longer time to conceive and more fertility problems than *T. gondii*-negative women (40).

This study was the first molecular detection of *T. gondii* in fetoplacental tissues of women with RSA, however it had some limitations. We did not access the previous abortion samples of the patients. In addition, we were unable to follow the patients and their future pregnancies. Hence, our study only suggested that toxoplasmosis might play a role in the pathogenesis of RSA. Additional investigations with larger groups of patients should be conducted in order to elucidate a clear relationship between *T. gondii* infection and RSA.

## Conclusion

The results of this study have indicated that genotype III is the predominant type of *T. gondii* in women with RSA in Tehran, Iran. Our results also indicated that *T. gondii* infection might play a role in the pathogenesis of RSA. However, more research should be conducted in this regard to elucidate a clear relationship between *T. gondii* infection and RSA.
